# Advanced Fault Diagnosis and Health Monitoring Techniques for Complex Engineering Systems: 2nd Edition

**DOI:** 10.3390/s25227054

**Published:** 2025-11-19

**Authors:** Yongbo Li, Teng Wang, Khandaker Noman, Bing Li

**Affiliations:** 1 School of Aeronautics, Northwestern Polytechnical University, Xi’an 710072, China; yongbo@nwpu.edu.cn (Y.L.); wangteng@nwpu.edu.cn (T.W.); bingli@nwpu.edu.cn (B.L.); 2 School of Civil Aviation, Northwestern Polytechnical University, Xi’an 710072, China

Health monitoring and advanced fault diagnosis are now critical features in modern engineering systems, the increasing complexity and new frameworks of which elevated the pressure for dependable reliability and safety measures. Since their inception, relevant techniques have evolved beyond their conventional use in aeronautical, civil and mechanical engineering and have become valuable tools for detecting faults early on in their development and for projecting the remaining useful life (RUL) of elements. Their application allows the timely execution of maintenance interventions, thus avoiding catastrophic failures and maximizing resource allocation as well as lowering downtime. The contemporary methods of diagnosis also promote better structural behavior, knowledge, performance and reduced maintenance. With these developments as the focus, this Special Issue features sixteen research papers that demonstrate new procedures and applications in advanced fault detection and health measurements of complex engineering systems.

In their work, Kucera et al. proposed the application of electronic fuel injectors in spark-ignition engines. A finite magnetic circuit model was developed to account for reluctance, inductance, resistance and magnetic flux (Contribution 1) and experiments were conducted using a programmable ECU, launchpad controller and a pressure vessel covering 0–300 kPa. The results showed that waveform inflection points reliably indicated needle motion. Injector performance was found to be affected by mechanical wear, pressure fluctuations and resistance variations, which led to delayed needle lift, reduced peak currents and waveform distortions. An automated tool to compare measured and reference signals was proposed to provide a non-invasive, fast and effective method for enhancing engine reliability and supporting preventive maintenance.

A hybrid CBAM-TCN-SVM framework for gear fault detection was proposed by Huang et al. and performed well with limited data (Contribution 2). In the proposed method, the vibration signals were first processed in terms of frequency-domain characteristics through Fast Fourier Transform (FFT), with an enhanced temporal convolutional network (TCN) with convolutional block attention modules (CBAM) employed to extract the important temporal and spatial information in the processed signals. These characteristics were then categorized with the help of an optimized support vector machine (SVM), which provided a possibility to identify different gear conditions correctly. The framework displayed an overall accuracy of 98.3% during the application for four gear states. The improved performance and notable robustness of this hybrid methodology were confirmed by further validation as well as through comparisons with conventional fault detection methods, confirming its suitability for application in the predictive maintenance of industrial systems, especially in environments where there are limited datasets, to support early fault acquisition and improved operational efficiency.

Utilizing the FRF-based model of optimal sensor placement for structural health monitoring, Kim et al. aimed to use the effective deployment of sensors to optimize structural observability and reduce measurement redundancy (Contribution 3). The methodology combined four criteria, namely mean kinetic energy, mean strain energy, modal assurance and mutual information, with optimization techniques such as greedy, genetic algorithm (GA), particle swarm optimization (PSO) and simulated annealing (SA). Validation was performed on a ten-story, three-bay shear-building model using Udwadia–Kalaba (U–K) constrained FRF expansion to reconstruct system responses from limited measurements. Monte Carlo simulations with model-driven noise showed that energy-based criteria produce focused high-fidelity layouts, while modal assurance and mutual information yielded globally informative sensor distributions. Jaccard similarity analysis confirmed the scalability, stability and robustness of the approach in measuring noise, emphasizing the practicality of its employment in real-world SHM systems.

Neural networks were employed by Rychlik to detect defects in light-alloy wheel rims using acoustic parameters such as reverberation time, absorption coefficient and total energy (Contribution 4). Laboratory experiments, FEM simulations and operational data revealed that reverberation time and absorption coefficient were the most reliable indicators of rim condition. A feedforward neural network trained on “Wheel Resistance Test Rig (WRTR)” data achieved high diagnostic accuracy that was also generalized well to real-world scenarios, with additional tests with mechanically damaged rims demonstrating the ability of neural networks to detect borderline faults. Their work also emphasizes the suitability of this low-cost non-contact approach for use in routine inspections and real-time wheel monitoring.

The integration of Health Indicators (HIs) and Digital Twins (DTs) for the predictive maintenance of rotating machinery was explored in detail by Bublil et al. (Contribution 5). The importance of proper labeling via seeding and endurance tests was highlighted for both physics-based and data-driven approaches. Auto-encoders and Generative Adversarial Networks (GANs) were applied to detect anomalies, while physics-based methods such as oil spill detection verified the results. By combining AI techniques with physical models, they found that fault classification and severity estimation were enhanced. Using Bayesian inference and Monte Carlo simulations to quantify uncertainty, they concluded that combining HIs with DTs in a single application improves predictive maintenance strategies and operational efficiency.

Lu et al. introduced a SAN-LSTM model for predicting aircraft engine vibration signals. While L1 filtering optimized slice lengths and tail amendments addressed incomplete segments, automatic recognition of operational states improved the model’s input for more accurate predictions (Contribution 6). Comparative tests against SAN+DLinear, transformer and informer models showed lower RMSE and MAPE for SAN-LSTM, highlighting the model’s ability to provide reliable trend forecasting, early anomaly detection and robust planning for aircraft maintenance schedules.

Cheng et al. proposed a real-time assessment model that could evaluate ground insulation degradation in the traction drive systems of locomotives and high-speed trains. To make it reliable and flexible, this framework was tested on five operating conditions and six different fault types (Contribution 7). When the insulation resistance dropped to less than 20k, distinct patterns of indicators were formed, with consistent monotonic changes in the voltage and detection measures as a function of this specific fault type. Other forms of faults had square-wave signal characteristics with growing variance, requiring the use of centroid-based decision rules to classify faults correctly. The system had low error cost and high accuracy of detection despite having some minor short-term misclassifications. The study also highlighted that the framework can be conveniently incorporated into the current vehicle controllers, providing a realistic and informative remedy for real-time railway monitoring and maintenance.

A deep transfer learning method was applied by Zhao et al. for bearing fault diagnosis across machines and operating conditions (Contribution 8). Sliding window segmentation combined with a ResFCN network was incorporated and fine-tuned on limited target datasets. Over 98% accuracy, recall and F1-score were achieved on sixteen transfer tasks using Case Western Reserve University (CWRU) and Paderborn University (PU) datasets. The framework also maintained consistent performance across variations in load, torque and sensor placement, avoiding the negative transfer and class imbalance issues seen in traditional methods. This shows the potential of the model for robust deployment in industrial settings with varying machine conditions.

The motor current signature analysis (MCSA) framework, enhanced with wavelet decomposition and autoregressive (AR) spectral modeling for motor-driven systems, was proposed by Diversi et al. (Contribution 9). Daubechies wavelets decomposed current signals and entropy differences across detailed levels served as fault indicators, while AR modeling produced power spectral densities and the symmetric Itakura–Saito spectral distance (SISSD) quantified deviations from healthy states. This approach was shown to be computationally efficient, making it suitable for edge computing and PLC-based monitoring without requiring detailed physical modeling of components.

Jiang et al. investigated compound fault detection in wind turbine gearboxes using a hybrid approach combining a Modified Signal Quality Coefficient (MSQC) and a Versatile Residual Shrinkage Network (VRSN) (Contribution 10). Vibration signals from operational turbines were denoised using MSQC to preserve fault-sensitive features. The dual-branch VRSN predicted the number of faults and probability distributions without manually set thresholds. The dataset included tooth wear, misalignment and bearing defects. The proposed approach achieved an average diagnostic accuracy of 96.26%, demonstrating computational efficiency suitable for real-time monitoring and minimizing turbine downtime.

Fault monitoring of variable-speed direct-drive marine current turbines (MCTs) under harsh marine conditions was achieved by Zhang et al. using water tanks, stabilizers and multiple sensors (Contribution 11). Stator voltage and current signals were recorded and fault-relevant features were extracted using the Matrix Pencil Method (MPM), which outperformed conventional spectral techniques. Generalized likelihood ratio tests combined with data normalization enabled the detection of small imbalances (1–3%). This methodology allows reliable long-term monitoring and preventive maintenance, improving energy production efficiency and turbine lifespan.

An Adaptive Resampling Optimization Support Vector Machine (ARO-SVM) framework was developed by Ai et al. for detecting reverse-connection defects in high-voltage cable cross-bonded grounding systems (Contribution 12). Power System Computer-Aided Design (PSCAD) simulations modeled cable length, burial depth and load resistance, generating 770 datasets. Amplitude and phase features were extracted and used as an input to the ARO-SVM classification model for optimized sampling. The framework accurately classified healthy and faulty states and outperformed conventional SVMs. Furthermore, considering its robustness and suitability during real-time power transmission monitoring, the proposed method reduces the risk of catastrophic cable failures.

Li et al. developed a Digital Twin-based framework for fault diagnosis in marine fuel systems. The system was designed to replicate real engine behavior under seven operating conditions, including injection delays, pump leakages and turbocharger failures (Contribution 13). In the digital environment, a Siamese Vision Transformer (SViT) was used to extract key diagnostic features from sensor data. These characteristics were then classified using the similarity-weighted k-nearest neighbor (KNN) algorithm to enable precise fault detection with a small number of labeled samples. To improve data quality during preprocessing, the researchers employed wavelet denoising and normalization. The model achieved a classification accuracy of more than 97% across various fault cases, showing the Digital Twin simulation and SViT-based learning approach to be effective in their application to marine engines for the tasks of real-time health monitoring and predictive maintenance.

In their work, Alharbi et al. proposed a chroma-enhanced semi-supervised anomaly detection (CASSAD) system for industrial anomaly detection of conveyor belt idler tracking (Contribution 14). Combining spectral and temporal characteristics with the help of Constant-Q Transform (CQT), Short-Time Fourier Transform (STFT) and Chroma Energy Normalized Statistics (CENS), CASSAD needed very few labeled samples in contrast to the traditional systems, which were supervised. Experimental tests yielded an area under the curve (AUC) score of 0.96, indicating that the system’s high sensitivity and resilience, even in noisy conditions. Together, receiver operating characteristic (ROC), precision-recall curve visualization and t-SNE mapping proved a clear distinction between normal and faulty states, presenting CASSAD as a computationally efficient, scalable system that can be applied for real-time industrial monitoring.

Liu et al. proposed a hybrid diagnostic framework combining Markov Transition Fields and a Squeeze-and-Excitation Residual Network to identify faults in planetary gearboxes (Contribution 15). In their study, one-dimensional vibration data were transformed into two-dimensional representations that preserved both temporal and frequency information. The SE-ResNet34 model analyzed these representations to highlight fault-related features and suppress irrelevant noise, with experimental validation across multiple load conditions achieving a diagnostic accuracy of 98.1%, surpassing conventional deep learning architectures such as CNN and ResNet. Therefore, regarding the issue of predictive maintenance in complex mechanical systems, the hybrid approach presented in their research provides a suitable, reliable and accurate diagnostic solution.

In Leite et al.’s extensive review of 29 publications investigating the use of machine learning for the detection of faults in Industry 4.0, several key issues were revealed such as the low quality of acquired data, the lack of model interpretability and the inability to integrate diagnostic tools into the current industrial environments (Contribution 16). Though it is observed that some of the supervised and unsupervised methods, such as decision trees, support vector machines (SVMs) and deep neural networks, have delivered promising results in experimental research, their applications in actual industry contexts are limited by the issue of scalability and transparency. To overcome this, they suggest the creation of standard benchmark datasets and the use of effective techniques to resolve the issue of class imbalance. Moreover, they argue that it is necessary to model the temporal features to increase the accuracy of diagnoses, also highlighting the significance of Explainable AI (XAI) models as tools with which to improve model explainability.

The editors express their sincere gratitude to all the contributing authors in this issue for their significant contributions to the field of fault diagnosis and health monitoring. Special thanks are extended to the reviewers for their constructive feedback, which has greatly enhanced the overall quality of this Special Issue. Finally, deep appreciation is extended to the editorial board of *Sensors* for their continued support in facilitating the dissemination of these important works.

